# 3D printed polylactic acid/gelatin-nano-hydroxyapatite/platelet-rich plasma scaffold for critical-sized skull defect regeneration

**DOI:** 10.1186/s12938-022-01056-w

**Published:** 2022-12-12

**Authors:** Marjan Bahraminasab, Nesa Doostmohammadi, Athar Talebi, Samaneh Arab, Akram Alizadeh, Ali Ghanbari, Amir Salati

**Affiliations:** 1 Nervous System Stem Cells Research Center, Semnan University of Medical Sciences, Semnan, Iran; 2 Department of Tissue Engineering and Applied Cell Sciences, School of Medicine, Semnan University of Medical Sciences, Semnan, Iran; 3 Faculty of Metallurgical and Materials Engineering, Semnan University, Semnan, Iran; 4 Research Center of Physiology, Semnan University of Medical Sciences, Semnan, Iran

**Keywords:** Bone regeneration, Platelet-rich plasma (PRP), 3D printing, Gelatin, Nano-hydroxyapatite

## Abstract

**Background:**

Three-dimensional (3D) printing is a capable approach for the fabrication of bone tissue scaffolds. Nevertheless, a purely made scaffold such as polylactic acid (PLA) may suffer from shortcomings and be restricted due to its biological behavior. Gelatin, hydroxyapatite and platelet-rich plasma (PRP) have been revealed to be of potential to enhance the osteogenic effect. In this study, it was tried to improve the properties of 3D-printed PLA scaffolds by infilling them with gelatin-nano-hydroxyapatite (PLA/G-nHA) and subsequent coating with PRP. For comparison, bare PLA and PLA/G-nHA scaffolds were also fabricated. The printing accuracy, the scaffold structural characterizations, mechanical properties, degradability behavior, cell adhesion, mineralization, systemic effect of the scaffolds on the liver enzymes, osteocalcin level in blood serum and in vivo bone regeneration capability in rat critical-sized calvaria defect were evaluated.

**Results:**

High printing accuracy (printing error of < 11%) was obtained for all measured parameters including strut thickness, pore width, scaffold density and porosity%. The highest mean ultimate compression strength (UCS) was associated with PLA/G-nHA/PRP scaffolds, which was 10.95 MPa. A slow degradation rate was observed for all scaffolds. The PLA/G-nHA/PRP had slightly higher degradation rate, possibly due to PRP release, with burst release occurred at week 4. The MTT results showed that PLA/G-nHA/PRP provided the highest cell proliferation at all time points, and the serum biochemistry (ALT and AST level) results indicated no abnormal/toxic influence caused by scaffold biomaterials. Superior cell adhesion and mineralization were obtained for PLA/G-nHA/PRP. Furthermore, all the developed scaffolds showed bone repair capability. The PLA/G-nHA/PRP scaffolds could better support bone regeneration than bare PLA and PLA/G-nHA scaffolds.

**Conclusion:**

The PLA/G-nHA/PRP scaffolds can be considered as potential for hard tissue repair.

## Background

As a typical complex tissue, bone has a hierarchical structure serving several functions including supporting the body and interior organs, movement simplification, saving and releasing minerals [1]. Therefore, bone defect is a critical illness, and health problem that leads to arthritis, neoplasm, osteoporosis, and congenital defects [2]. Among the treatments, bone grafts (allografts and autografts) have some advantages like osteoinductivity and osteoconductivity. But due to some drawbacks, such as high operative time and cost, the possibility of rejection, disease transfer risk, the lack of a transplant source, and chronic pain, attempts are being made to find alternative treatments over the bone graft minerals [3–6].

To overcome the disadvantages of current treatments, bone tissue engineering (BTE) has developed a method based on biomaterials as tissue scaffolds [7]. Designing and making a suitable scaffold made of synthetic and/or natural polymers with similarity to the biological, structural, chemical and mechanical characteristics of innate bone is the most vital part of the BTE. Since using scaffold has significant features such as high surface-to-volume ratio, resemblance to the extracellular matrix (ECM), low-cost production, and flexibility in the fabrication process, it has attracted enormous interest [8, 9]. Because of customized and accurate architecture, structures with interconnected pores, and manageable sizes and shapes of three-dimensional (3D) printed scaffolds, acceptable cell growth and proliferation are achievable in vitro and in vivo [10, 11].

Nowadays, most polymers used are polyesters (like polycaprolactone (PCL), polylactic acid (PLA)), and polylactic-co-glycolic acid (PLGA), and hydrogels (like alginate, gelatin, and hyaluronic acid) [12, 13]. PLA is a popular biodegradable polymer having a broad range of applications such as the fabrication of medical implant devices, and scaffolds in tissue engineering. The broad use of PLA is due to a combination of favorable properties including its easy processability, unique biocompatibility, acceptable bioresorbability rate which matches with the tissue healing time of injured human bones, generation of nontoxic byproducts during the degradation in the body, and approved clinical trials by the United State Food and Drug Administration (US FDA) [14, 15]. The only drawback associated with the use of PLA is the absence of the surface property to enable cell adhesion on its surface and the subsequent proliferation [16, 17]. Initial attachment of cells on the surface is the most primary criterion for the materials used to make implantable scaffolds, which substantially influences the biological responses. Poor interaction of cells with material surface results in unsuccessful scaffold function. Therefore, in recent years, a large number of biomaterials has been incorporated into PLA structures to provide improved properties [18–21].

Gelatin is a natural biopolymer with superb biocompatibility and biodegradation properties, containing ligands (binding receptors) that can enhance cell adhesion, differentiation, and proliferation [22, 23]. Alternatively, hydroxyapatite (HA) is a popular biomaterial for bone regeneration due to its outstanding bioactivity, acceptable mechanical strength, osteoconductivity, angiogenic properties, non-toxicity, and non-inflammatory reactions, particularly in nanoscale [24, 25]. The incorporation of these materials into 3D printed PLA may help in providing efficient cell interactions and new bone formation. Furthermore, it has been indicated that the use of bio-factors including growth factors (such as vascular endothelial growth factor (VEGF)) along with PLA scaffolds can promote bone regeneration [7, 26]. Platelet-rich plasma (PRP) contains growth factors that strongly affect the cell–extracellular matrix communication within the regeneration process [27]. Since PRP releases growth factors from alpha granules in platelets, it has the ability to enable healing of the damaged tissue, treat cartilage and osseous defects in periodontal regenerative therapy, and can possibly stimulate the proliferation and differentiation of mesenchymal stem cells (MSCs) in bone marrow [28–30].

In the present study, therefore, the aim was to fabricate PLA scaffolds by a 3D printing method, to infill the PLA scaffolds with gelatin-nanoHA, and to coat them with PRP. The structural, physical, and mechanical properties of the scaffolds were examined. Furthermore, the viability and proliferation of pre-osteoblast MC3T3-E1 cells exposed to scaffold culture media were evaluated by MTT assay. To a more detailed evaluation of the osteoinductivity of the scaffolds, they were implanted in the rat critical-sized calvaria defects and the bone regeneration was analyzed 8 and 12 weeks after implantation using histological analysis.

## Results

### Printing accuracy and physical properties

The printing accuracy was assessed by comparing strut thickness, pore width, density and porosity% of the PLA printed scaffolds with those defined in the computer-aided-design (CAD) model. The results are shown in Table 1. As can be seen, the mean printing errors were very low (all below 11%). The printed strut thickness was slightly higher (3.23%) than the defined value (400 μm), which caused the pore width to decrease (739.5 μm vs. 800 μm). Therefore, the printed density was higher and the porosity was lower compared to the defined values in the CAD model. The mean density and porosity% of PLA scaffolds were 0.426 g.cm^3^ and 65.68%, respectively.

**Table 1:** Comparison of the geometry, density and porosity of the printed scaffolds with the CAD model

	Strut thickness	Pore width	Density	Porosity
Printed PLA	412.9 ± 21.4 (μm)	739.5 ± 13.6 (μm)	0.426 ± 0.006 (g cm^3^)	65.68 ± 0.454 (%)
CAD model	400 (μm)	800 (μm)	0.385 (g cm^3^)	68.99 (%)
Mean printing error (%)	3.23	− 7.56	10.66 (%)	− 4.79 (%)
Printing error range (%)	− 3.5–8.25	(− 9.9)–(− 4.9)	8.3–16.14	(− 7.3)–(− 3.8)

### Morphology of nHA powder and the scaffolds

**Fig. 1:**
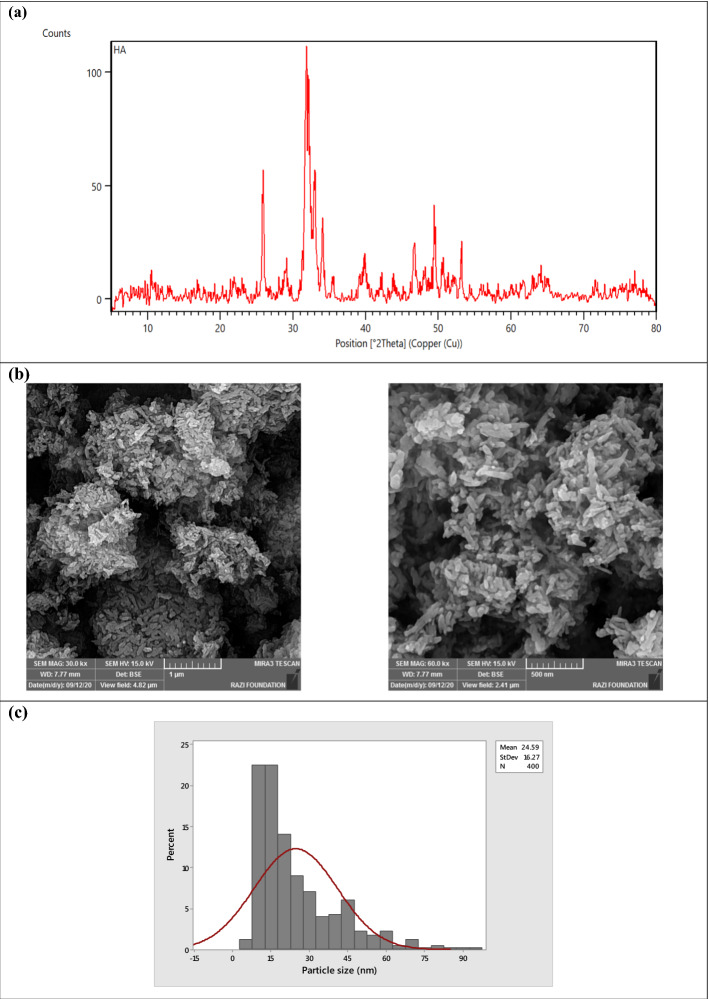
Characteristics of the nHA powder used

Figure 1 shows the X-ray diffraction (XRD) pattern, scanning electron microscopy (SEM) images and the size distribution of the nHA powder. The XRD reflections of nHA powders match with reference cards of 01-1008 and 46-0905 which are related to hydroxyapatite (Ca_10_(PO_4_)_6_(OH)_2_) [31] and calcium-deficient hydroxyapatite (Ca_9_HPO_4_(PO_4_)_5_OH) [32]. The HA triplet peaks appeared at 2*θ* around 31.8°, 32.2°, and 32.9° were diffracted from the planes of (211), (112), and (300), respectively. Other peaks at 33.9 and 39.81 (planes (202) and (310)) and some minor reflections in the 2θ range from 40–55° were also detected. Similar peaks have been reported previously in the studies of HA synthesis [25, 33, 34]. SEM images showed that the nHA particulates have an irregular shape (Fig. 1b) with an average size of 24.59 (± 16.27) nm. Figure 1c represents the powder size distribution, which was < 100 nm.

**Fig. 2:**
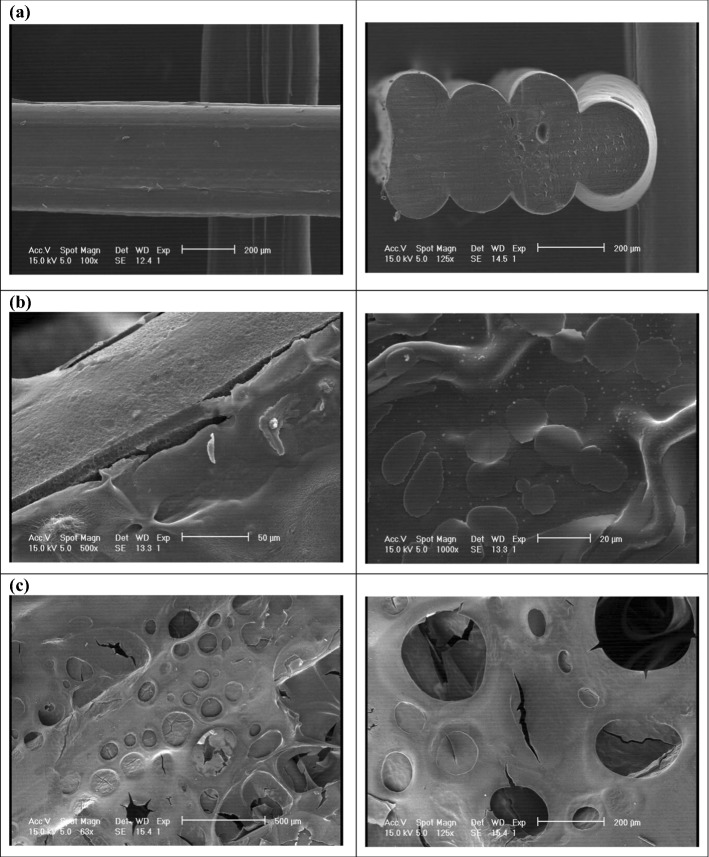
SEM images of scaffolds: **a** PLA, **b** PLA/G-nHA, and **c** PLA/G-nHA/PRP

Furthermore, the surfaces of PLA, PLA/G-nHA, and PLA/G-nHA/PRP scaffolds were observed by SEM (Fig. 2). The surface of the PLA strut and the cross section of a PLA scaffold are shown in Fig. 2a. The G-nHA-infilled scaffolds are shown in Fig. 2b. The G-nHA filled the pores of the scaffold and connected the struts. However, some micro-cracks were observed at strut sites (Fig. 2b, left image), possibly due to the shrinkage occurred during the freeze-drying. The surface of G-nHA also can be seen in the right image of Fig. 2b. Furthermore, the surfaces of a PLA/G-nHA/PRP scaffold can be seen in Fig. 2c, where PRP completely coated the surface of the PLA/G-nHA scaffold. Several pores and small cracks can be observed on the PRP surface because of freeze-drying.

**Fig. 3:**
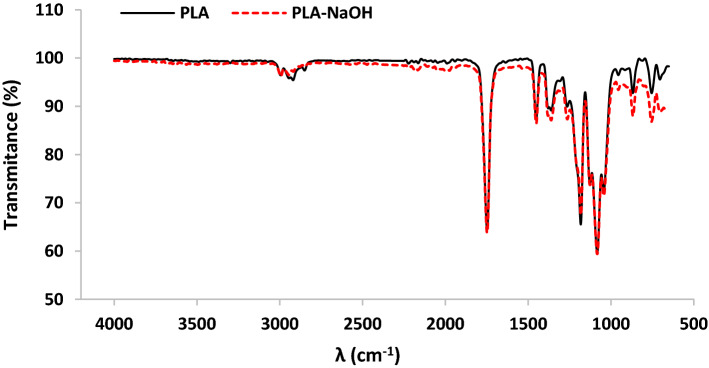
FTIR spectra of PLA and NaOH-treated PLA scaffolds

The attenuated total reflection-Fourier transform infrared (ATR-FTIR) spectra of PLA scaffold before and after treatment by NaOH are shown in Fig. 3. The characteristic bands at about 2994 cm^−1^ and 2944 cm^−1^ are related to asymmetric and symmetric –CH stretching in –CH_3_ group, respectively. Furthermore, the peak appeared at 2922 cm^−1^ is attributed to –CH bending vibration. In the ATR-FTIR spectrum of the PLA scaffold, peaks were also detected at 756 and 867 cm^−1^ (–CH bending vibration); 1043 cm^−1^ and 1182 cm^−1^ (C–O stretching vibration); 1082 cm^−1^ (stretching peaks of C–O–C bonds) 1451 cm^−1^ and 1361 cm^−1^ (asymmetric and symmetric –CH bending in –CH3 group, respectively); and 1748 cm^−1^ (C=O stretching vibration on ester group)**.** The observed peaks correspond to PLA and agree well with data in the previous studies [35–37]. Furthermore, the same peaks appeared on the ATR-FTIR spectrum of PLA after NaOH treatment with negligible difference in intensity (dash red line in Fig. 3).

### Mechanical properties

**Fig. 4:**
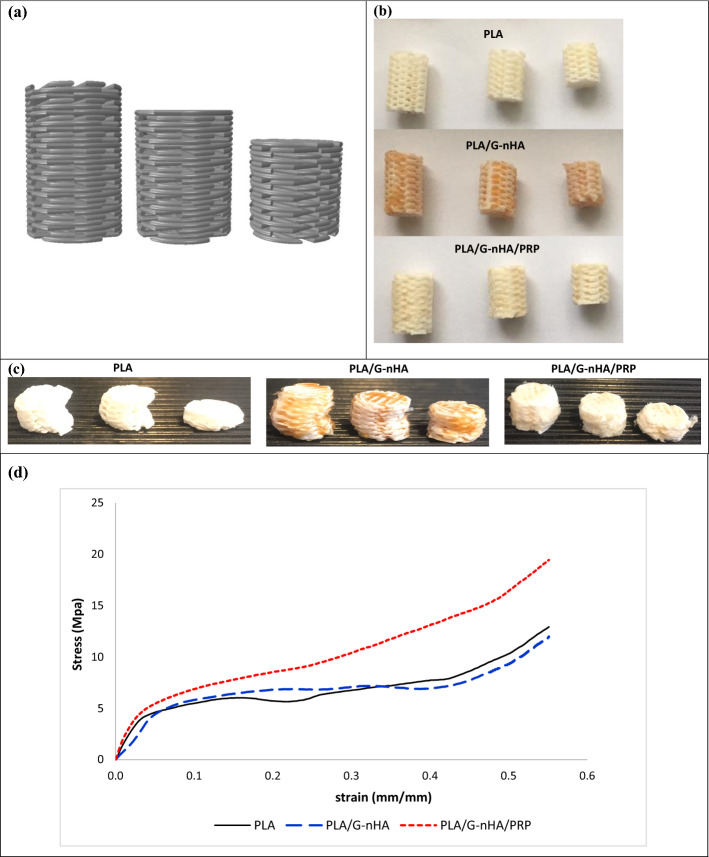
**a** and **b** CAD model and actual compression specimens, **c** compressed samples, and **d** stress–strain curve

Compression tests were done to indicate the highest compressive load carrying ability of the fabricated scaffolds. The results indicated three distinct areas in the stress–strain curve obtained from compressive test including linear elasticity, long plateau, and densification region, for all PLA, PLA/G-nHA, and PLA/G-nHA/PRP scaffolds (Fig. 4). The elastic moduli (E) were estimated from the initial linear region, and ultimate compressive strengths (UCS) were also determined. The results are presented in Table 2. The UCS was highest for PLA/G-nHA/PRP scaffolds (10.95 MPa), followed by PLA/G-nHA scaffolds (6.62 MPa). The PLA scaffolds had the lowest UCS (5.15 MPa). The pores on 3D printed PLA scaffolds filled by G-nHA and PRP possibly caused higher compressive strengths for these scaffolds. The values of E were highest for PLA, which was 241.18 MPa. However, the PLA/G-nHA and PLA/G-nHA/PRP scaffolds were less stiff (121.69, and 225.29 MPa, respectively).

**Table 2:** Mechanical properties of the scaffolds

Materials	Weights (mg)	UCS (MPa)	E (MPa)
PLA	25.5 ± 0.34	5.15 ± 1.8	241.18 ± 54.7
PLA/G-nHA	30.7 ± 0.88	6.62 ± 0.5	121.69 ± 30.0
PLA/G-nHA/PRP	34.0 ± 0.93	10.95 ± 1.6	225.29 ± 39.3

### Biodegradability behavior

**Fig. 5:**
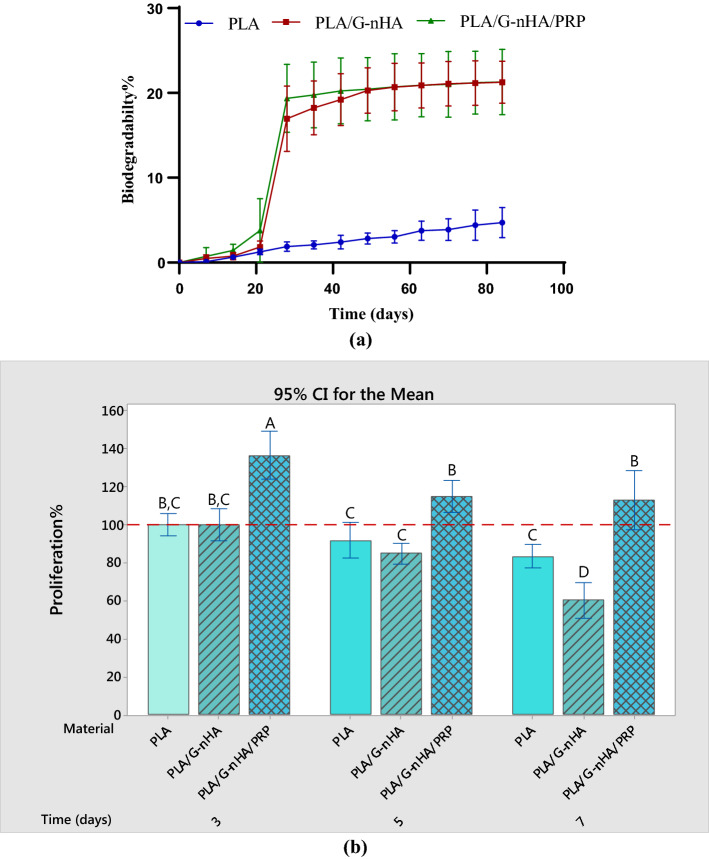
**a** Biodegradation behavior of scaffolds, and **b** cell proliferation

The in vitro biodegradation test was performed by immersion of three scaffolds of each group individually in simulated body fluid (SBF) solution for 12 weeks (84 days) in a thermostatic oven at 37 ± 0.5℃. The percentages of weight changes were calculated every 7 days based on Eq. 2. The results are shown in Fig. 5a which indicate the faster degradation of PLA/G-nHA/PRP scaffolds, likely because of the PRP coating on the surfaces. After 21 days, the degradation of 1.3%, 1.8% and 3.8% was obtained for PLA, PLA/G-nHA, and PLA/G-nHA/PRP, respectively. However, during week 4 (21–28 days), a sudden increase in weight loss was observed for PLA/G-nHA, and PLA/G-nHA/PRP which was due to the release of G-nHA and PRP to the solution. After that, the biodegradation rates were very slow.

### Cell proliferation

MC3T3-E1 cell proliferation exposed to the culture media of the fabricated scaffolds was assessed using MTT assay after 3, 5, and 7 days. Figure 5b shows the proliferation% for the studied scaffolds. As can be seen, at day 3 of the cell exposure to the scaffold extract medium, the proliferation rates were higher compared to days 5 and 7 in all groups. The maximum proliferation% was related to PLA/G-nHA/PRP scaffolds, which was about 136.2% at day 3. However, the proliferation percentages of the cells exposed to extract medium of PLA/G-nHA/PRP scaffolds reduced to 114.8% and 112.8% at days 5 and 7, respectively. Statistical analysis (two-way analysis of variance (ANOVA)) determined that both scaffold material and time (*P*-values of 0.000), and their interaction effect (time* materials, *P*-value of 0.009) were significant factors in cell proliferation%. Tukey pairwise comparison was also done and shown in Fig. 5b. The means that do not share a letter are significantly different. Therefore, the proliferation% associated with PLA/G-nHA/PRP scaffolds was significantly higher than those of other groups at each time point. Furthermore, the proliferation% after 3 days was significantly different from those obtained after 5 and 7 days for PLA/G-nHA/PRP scaffolds. However, the difference between proliferation% related to PLA and PLA/G-nHA scaffolds at days 3 and 5 were not statistically significant.

### Adhesion of cells to the scaffolds

**Fig. 6:**
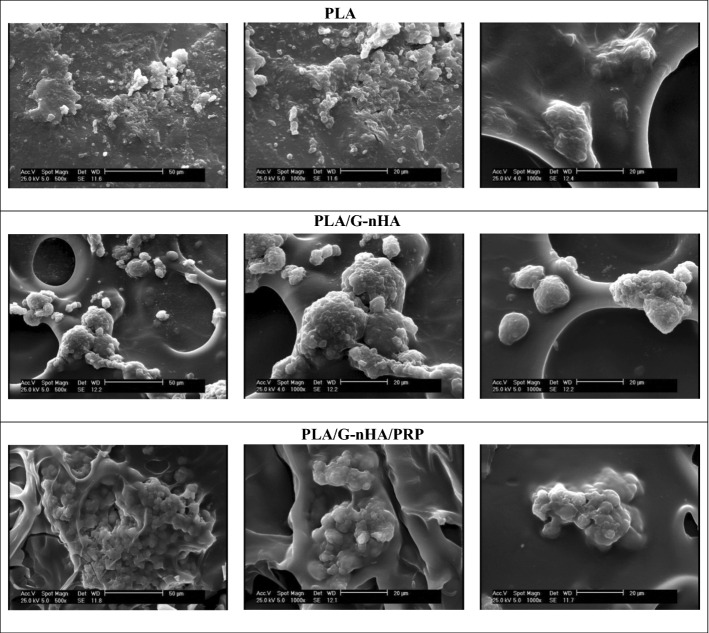
SEM images of the adhered cells on different scaffolds

The capability of the scaffolds on adhering MC3T3-E1 cells after 3 days of culture was analyzed by SEM images (Fig. 6). The number of adhered cells on different scaffolds was different. The cells were quite few on the PLA scaffolds. However, the adhered cells were high in PLA/G-nHA and PLA/G-nHA/PRP scaffolds. As can be seen in Fig. 6, the cells attached and grew better on the surfaces of these scaffolds. It appears that the cells attached to the pore walls or on the open pores on the PLA/G-nHA and PLA/G-nHA/PRP scaffolds. This confirms that the bioactive materials (gelatin and PRP) and their surface topography (freeze-dried induced pores) cause superior cell adhesion. Furthermore, larger cell aggregates were observed on the PLA/G-nHA/PRP scaffolds.

### Matrix mineralization

**Fig. 7:**
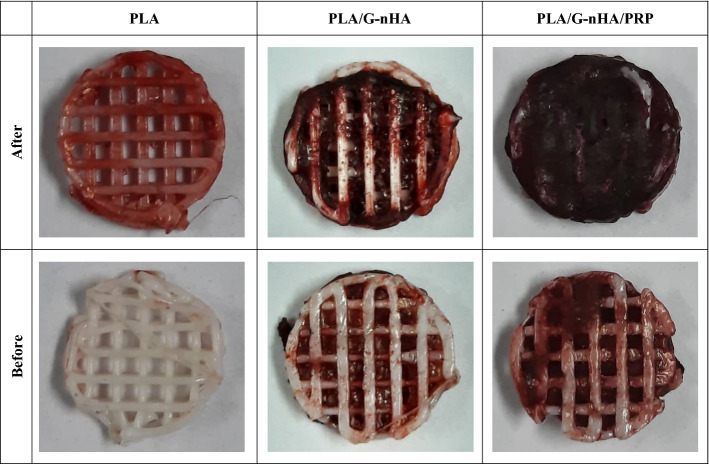
Images of the scaffolds stained by Alizarin Red before and after cell culture

The MC3T3-E1 cells were cultured in the osteogenic culture medium for 10 days to determine the mineralization and calcium deposit. Figure 7 shows the Alizarin Red staining of different scaffolds before and after cell culture. As can be seen, the mineralization and calcium deposition of MC3T3-E1 cells were occurred in all scaffolds. The red-stained area in PLA/G-nHA and PLA/G-nHA/PRP scaffolds before culture is related to the presence of hydroxyapatite. Nevertheless, the intensity and the area of the red color increased in these scaffolds, particularly in PLA/G-nHA/PRP group, meaning that the mineralization of cells were well occurred.

### Serum biochemistry

**Fig. 8:**
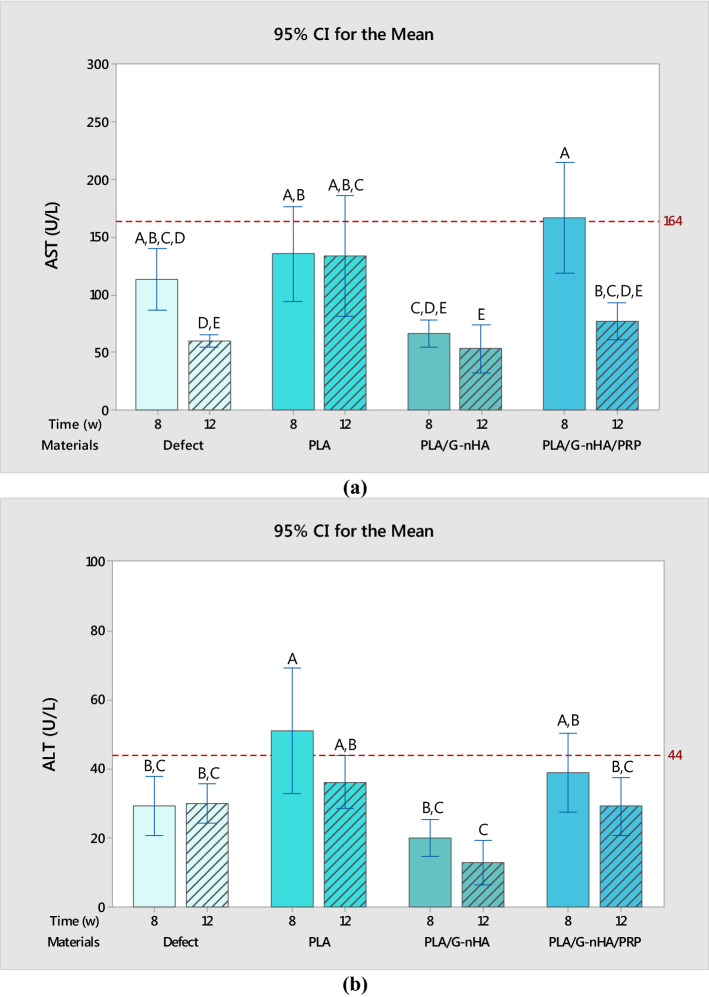
**a** ALT level, and **b** AST level in rat serum

The biochemical analyses (alanine aminotransferase (ALT) and aspartate aminotransferase (AST) level) were performed to monitor the systemic toxicity and any abnormal response possibly induced by the scaffolds. AST and ALT levels in serum are judiciously sensitive indicators of damage or injury to liver from various types of diseases or conditions. The ALT and AST levels in rat serum of different groups are shown in Fig. 8. The red lines in Fig. 8a and b indicate these enzyme levels for normal female rats with neither skull defects nor scaffold implantation. In the levels of both AST and ALT both materials (*P*-value of 0.000 for both AST and ALT) and time (*P*-values of 0.000 and 0.024 for AST and ALT, respectively) were influential. The means of AST levels almost in all groups of the scaffolds did not exceed the red line (in PLA/G-nHA/PRP group the mean value was slightly higher) meaning that the scaffolds did not cause systemic toxic effects. Similarly, the mean of the ALT levels did not surpass the normal level except for the rats with PLA scaffold implantation after 8 weeks. However, after 12 weeks the AST and ALT values decreased in all groups. Furthermore, the statistical differences among the groups in ALT and AST levels are also shown in Fig. 8a and b.

### Osteocalcin level in serum

**Fig. 9:**
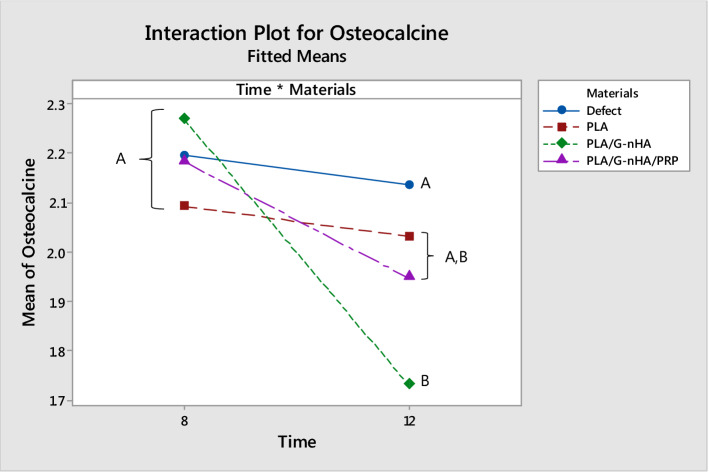
Mean ± STDV values of osteocalcin concentration in serum of rats (ng/mL)

The serum osteocalcin levels, shown in Fig. 9, were higher at week 8 than week 12 in each group. The lower level of this osteoblastic marker may be due to the fact that after 12 weeks the defects were mostly filled by new bone, particularly in PLA/G-nHA and PLA/G-nHA/PRP groups (Table 3). Furthermore, there were no significant differences in the serum osteocalcin levels among the groups 8 weeks after surgery. However, the level of osteocalcin in PLA/G-nHA group was significantly lower than in the defect group. Another issue is that the level of this marker in the defect control group was not change at the two time points. This is due to the fact that no new bone matrix was formed at the defect site in this group.

**Table 3:** Mean and STDV of the filled area by new bone in different groups

Groups	Time (week)	Filled area (%)
Defect	8	23.14 ± 1.40
	12	28.63 ± 1.30
PLA	8	58.58 ± 9.31
	12	71.08 ± 9.26
PLA/G-nHA	8	63.59 ± 6.47
	12	80.22 ± 7.57
PLA/G-nHA/PRP	8	69.80 ± 0.92
	12	83.68 ± 5.14

### Histological observation

**Fig. 10:**
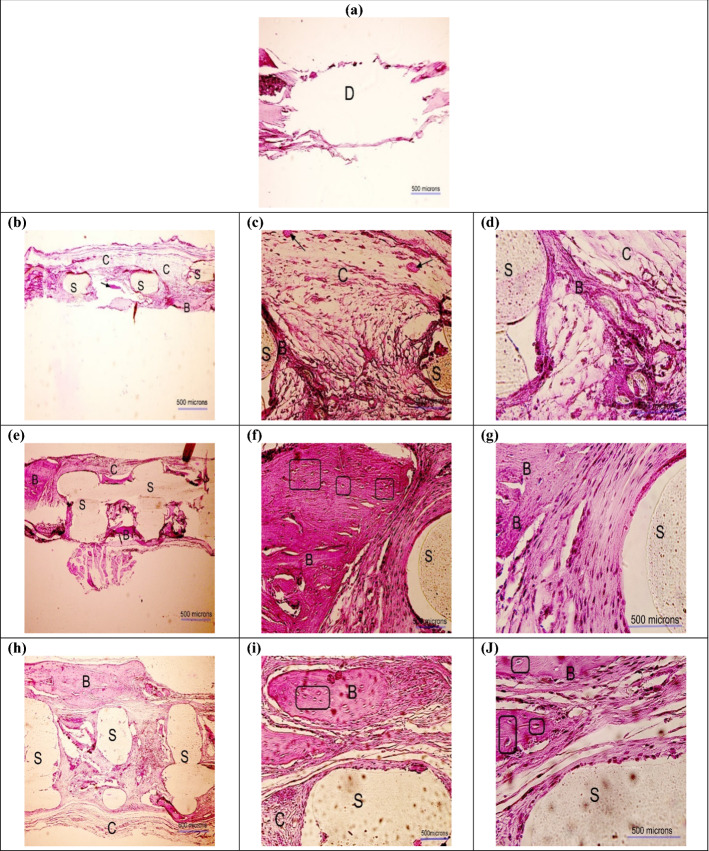
H&E images obtained 8 weeks postoperatively in different groups: **a** defect without scaffold, **b**–**d** PLA, **e**–**g** PLA/G-nHA, and **h**–**J** PLA/G-nHA/PRP. The scaffold, bone, connective tissues, and defect site are signified by "S", "B", "C", and "D", respectively. The new bone islands are indicated by black arrows and osteocytes are identified in rectangles. The magnifications are × 40, × 200, and × 400 in (**a**, **b**, **e** and **h**), (**c**, **f** and **i**), and (**d**, **g** and **j**), respectively

**Fig. 11:**
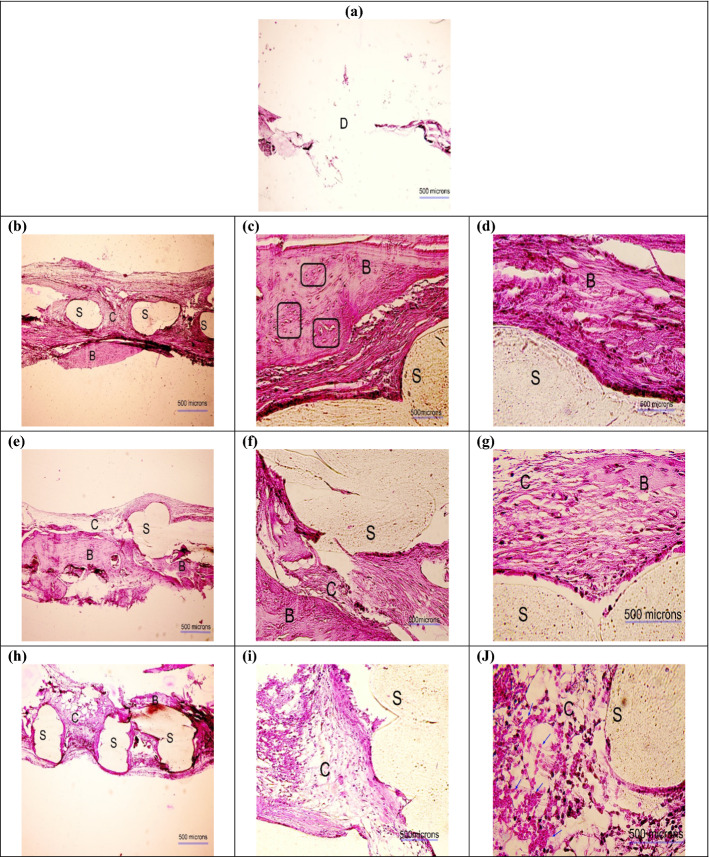
H&E images obtained 12 weeks postoperatively in different groups: **a** defect without scaffold, **b**–**d** PLA, **e**–**g** PLA/G-nHA, and **h**–**j** PLA/G-nHA/PRP. The scaffold, bone, connective tissues, and defect site are signified by "S", "B", "C", and "D", respectively. The new bone islands are indicated by black arrows and osteocytes are identified in rectangles. The magnifications are × 40, × 200, and × 400 in (**a**, **b**, **e** and **h**), (**c**, **f** and **i**), and (**d**, **g** and **j**), respectively

Figures 10 and 11 represent the histological images obtained after 8 and 12 weeks of implantation, respectively. In these figures, the scaffold is shown by the capital letter "S" (light beige color), the defect is identified by the capital letter "D", and the bone is marked by the capital letter "B" (pink color). The osteocytes are indicated by rectangles, and the new bone islands are pointed at by arrows. As can be seen in the histological images, the scaffolds in all groups still remained, as the biodegradability rate was slow (Fig. 5a). However, the new tissues including connective, and neo-bone were formed around the scaffolds in the pores. At the bone–scaffold interface, more mature bone was seen, while in the middle of the defect, the formed tissue was less strong. As can be seen in Figs. 10a and 11a, minimum bone formation was observed in the untreated defect/control group. Comparing the three scaffold groups, a higher degree of new bone growth and cartilaginous tissue was seen in the PLA/G-nHA and PLA/G-nHA/PRP groups rather than PLA. In the bare PLA group at week 8, most areas of the defect were occupied by connective fibrous tissue, and there was only small new bone. In the histological sections of PLA/G-nHA/PRP group, high vascularization and large number of red blood cells were observed. Furthermore, there was a higher new bone in the defect sites after 12 weeks in all groups. The morphology of normal bone was seen by the newly regenerated bone-like tissue with osteocytes embedded in bone matrix within their lacuna and osteoblasts lined the bone tissue exterior edge. The histological observation also revealed no inflammation macroscopically for all scaffolds. On a microscopic scale, however, small inflammation and presence of lymphocytes were observed in the PLA/G-nHA/PRP group. Table 3 shows the filled area of the defect by new bone in different groups. The values in this table were normalized to the defect size at the operation day. As can be seen, the filled area increased in all groups at week 12 compared to week 8. Furthermore, the filled area was highest for PLA/G-nHA/PRP group, followed by PLA/G-nHA. In the untreated defect (control) group, the filled area of the defects was mostly non-functional soft tissue, while in the scaffold groups, as shown by H&E staining, the filled area mainly had cartilaginous and bone tissues.

## Discussion

The main objective of the present study was to investigate the effect of incorporation of materials including gelatin, nano-hydroxyapatite and platelet-rich plasma on physical, mechanical and osteogenic properties of 3D printed PLA scaffolds. First, the scaffolds were printed based on a CAD model with very low printing error. Subsequently, by immersion and freeze-drying techniques, gelatin and nano-hydroxyapatite infilled the pores in the scaffolds, and finally the PLA/G-nHA scaffolds were PRP coated.

For bone tissue engineering applications, the amount of scaffold compressive strength is of importance. Nevertheless, tensile strength is vital for soft tissue replacements. Examples are the cartilage and skin substitutes, which function under tension and with flexibility. Among the studied scaffolds, PLA/G-nHA/PRP showed the highest strength followed by PLA/G-nHA. On the other hand, the PLA scaffolds were the stiffest with lowest flexibility. Mechanical properties of 3D printed PLA scaffolds have been investigated in different studies [38–42]. It has been indicated that the PLA scaffold elastic modulus was dependent on the molecular weight and the crystallinity degree of the PLA [43]. Furthermore, it has been indicated that the process parameters and layer orientation also influence the mechanical properties [39, 42]. Hence, different PLA fabricated scaffolds can show different mechanical properties. However, all PLA-based scaffolds in different study indicated three distinct areas in the compressive stress–strain curve (linear elasticity, long plateau, and densification region). Similarly, the scaffolds fabricated in the present study all demonstrated these three regions.

The biodegradation test, in the present study, showed that all scaffolds degraded over time. Once PLA comes in contact with biological solutions, it starts to break down, usually by hydrolysis of the ester-bond backbone [44]. The byproduct is lactic acid or is CO_2_ and H_2_O, which can be metabolized intracellularly or excreted in the urine and breath. Furthermore, other factors such as bacterial infection and foreign-body inflammation increase the PLA breakdown via the secretion of enzymes that degrade the polymer [45]. Our in vitro biodegradation behavior of the scaffolds demonstrated a low degradability rate. The bare PLA scaffolds were degraded only ⁓5% after 12 weeks of immersion in SBF. It has been indicated that PLA degradation rate can be influenced by a variety of factors including the shape and size of the material [46]. Therefore, studies that examined different forms of PLA in different conditions obtained dissimilar degradation rates. For example, implantation of a PLA sheet in the infraorbital rim of macaque monkeys showed that the fragments of PLA were found at the surgery site 38 weeks post-implantation, while PLA films in 0.2 M citrate buffer having pH 7 at 37 ℃ revealed 10% weight loss over 16 weeks [45]. The PLA/G-nHA and PLA/G-nHA/PRP scaffolds also showed low degradability which was slightly higher than bare PLA, possibly due to G-nHA and PRP release. However, a burst release of G-nHA and PRP occurred at week 3, and afterwards the degradation rate was similar to bare PLA.

The PLA and particularly PLA/G-nHA/PRP scaffolds showed high proliferation% at all time points. Several studies analyzed the cell proliferation for PLA scaffolds. For example, Fan et al. [47] fabricated electrospun PLA scaffolds which showed a similar proliferation percentage to our results (about 100% proliferation) after 3 days of exposure. Furthermore, the ALT and AST levels in rat serum showed no systemic toxicity after 12 weeks of implantation. Other studies on PLA and PLA-based scaffolds also showed ALT and AST levels within the normal limits in the serum at different time points and demonstrated good biocompatibility [48, 49].

Cell adhesion study showed that the cellular interactions were different on the scaffolds. This can be associated with the scaffold chemical composition, surface topography, and wettability [50, 51]. PLA is a hydrophobic material with high water contact angle [52] and it has been indicated that for achieving improved cell adhesion on PLA, surface modification or aid of bioactive materials are required [53, 54]. The result obtained on cell attachment in the present study also confirms this.

Bone mineralization occurs when the organic bone matrix fills with nanocrystals of calcium phosphates. Our results indicated higher mineralization of the MC3T3-E1 cells on PLA/G-nHA/PRP and PLA/G-nHA, respectively. This can be related to the use of bioactive materials including nHA, gelatin, and PRP.

Furthermore, osteocalcin level in rat serum was measured both at week 8 and 12 which was higher at week 8 compared with week 12. Osteocalcin is produced only by mature osteoblasts and plays a role in bone mineralization. It is mainly deposited into the bone extra cellular matrix and only a small quantity of its newly formed reaches the circulation [55, 56]. The lower level of this osteoblastic marker may be due to the fact that after 12 weeks the defects were mostly filled by new bone, particularly in PLA/G-nHA and PLA/G-nHA/PRP groups. In one study by Zhang and Zhang [56], the osteocalcin expression by MG63 cells exposed to microporous chitosan scaffolds reinforced by calcium phosphate was evaluated. Their results showed lower osteocalcin concentration at day 11 compared with that of day 7. This is in agreement with our findings.

The histological analysis showed that the new connective, and neo-bone tissues were formed around the scaffolds in the pores. At the bone–scaffold interface, more mature bone was seen, while in the middle of the defect, the formed tissue was less strong. The minimum bone formation was observed in the untreated defect/control group. Comparing the three scaffold groups, a higher degree of new bone growth and cartilaginous tissue was seen in the PLA/G-nHA and PLA/G-nHA/PRP groups rather than PLA. Previously reported findings, also demonstrated the efficiency of using PRP in bone regeneration. Abazari et al*.* showed that applying PRP as a bioactive agent, which was isolated from human blood, had a great potential to be used as an efficient bone implant in combination with polyvinyl-alcohol (PVA), chitosan, and hydroxyapatite nano-fibers [3]. In another study, the synergistic effects of HA and PRP on osteogenic activity were investigated [57]. The authors explained that HA/ZrO_2_ provides a suitable environment for the regeneration of the rabbit defect in the short term. While, the application of PRP and HA/ZrO_2_ had no synergistic effects that may be due to attribution to the species, gel versus liquid administration, and the method of HA preparation rather than the ineffectiveness of such a combination. Furthermore, a 3D nanofibrous PLLA-HA was developed by Koc et al. [58] and used for the irregular bone damage treatment. Then, PRP was applied into the surface modified grafts and activated. According to the results, 3D PLLA-HA had higher performance after using PRP. Moreover, the effect of using PRP coated composite 3D scaffolds on promoting osteogenic differentiation of adult stem cells in BTE was investigated by Wei et al*.* They showed that the PRP treatment highly upregulated the gene expression levels of late osteogenic markers [59]. Qiao et al. also fabricated 3D printed Ti6Al4V scaffolds coated with PRP as a bioactive interface. The porous titanium used in scaffolds had advantages of ideal porosity, pore size, and mechanical properties matching with those of bone tissue, thus, an artificial implant with a bioactive interface for patients with osteoporosis was successfully developed [60]. The results obtained here also confirm those reported previously.

It would be interesting for future research to further study the bone regeneration through other analyses such as micro-CT, a high-resolution imaging modality, which can accurately indicate the area/volume of newly formed bone and monitor the bone architecture during treatment.

## Conclusions

In the present study, PLA scaffolds were 3D printed, infilled by gelatin-nano-hydroxyapatite (PLA/G-nHA), and coated by PRP. The physical, mechanical, in vitro biological properties and in vivo bone regeneration were evaluated. The following could be concluded:

- High printing accuracy was achieved with the mean printing error of below 11% for all parameters including strut thickness, pore width, scaffold density, and porosity%.
- The highest mechanical strength (UCS) was obtained for PLA/G-nHA/PRP (10.95 MPa) scaffolds followed by PLA/G-nHA (6.62 MPa) and PLA (5.15 MPa), respectively.
- The degradation rate of all scaffolds was slow for which the PLA/G-nHA/PRP had slightly higher degradation, possibly due to PRP release. At week 4, burst release of G-nHA and G-nHA/PRP occurred.
- The PLA/G-nHA/PRP was shown to induce the highest cell proliferation at all time points, cell adhesion and mineralization.
- The serum biochemistry results rejected any abnormal or systemic toxicity caused by scaffold biomaterials.
- All the developed scaffolds showed bone regeneration capability, however there were differences between different scaffolds. PLA/G-nHA/PRP scaffolds possibly better support bone regeneration than bare PLA and PLA/G-nHA scaffolds.

## Materials and methods

### Fabrication of PLA scaffolds

**Fig. 12:**
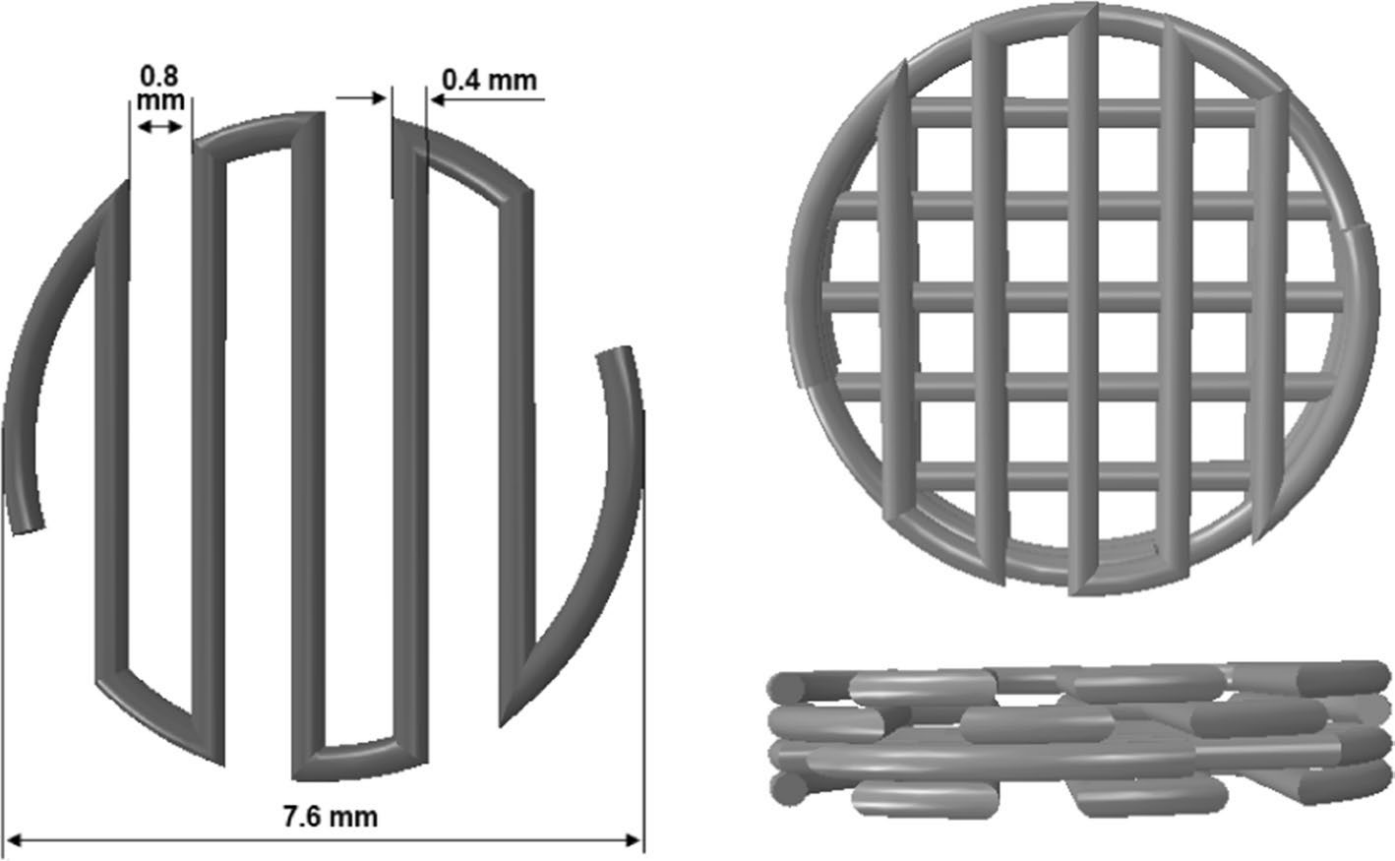
Designed CAD model of bone scaffolds

First, a 3D CAD model of the PLA scaffold was designed in ABAQUS software. Subsequently, the "stl." file format of the model was imported to Simplify3D software to provide the g-codes for manufacturing. Polylactic acid filament (diameter of 1.75 mm) was employed to build the scaffolds with a conventional fused deposition modeling (FDM) 3D printer. The PLA filament was heated above its melting point (nozzle temperature was 210 ºC). The melted PLA extrusion was done through a stainless-steel nozzle on to a printing bed heated at 60 ºC. The scaffolds were made in a layer-by-layer manner having a 7.6 mm diameter and a 1.6 mm height (Fig. 12).

### Preparation of PLA/G-nHA scaffolds

Gelatin (Merck, USA) and n-HA (Yazd Research Technology Co, Iran) powder were used to prepare the G-nHA suspension. To prepare PLA/G-nHA scaffolds, first gelatin was dissolved in distilled water to provide 10% w/v aqueous gelatin solution at 40 °C under rigorous stirring (400 rpm). When the gelatin solution became homogenous, nHA was gradually added to have 0.5% w/v suspension. The stirring was continued to obtain a uniform suspension. The fabricated PLA scaffolds were first immersed in distilled water for 1 h, and then rinsed and ultrasonicated in distilled water for 5 min. The clean scaffolds were immersed in NaOH 0.6 M for 6 h to provide surface roughness, and improve the hydrophilicity [61]. Finally, the NaOH-treated scaffolds were rinsed by distilled water (ATR-FTIR of the PLA scaffolds before and after NaOH treatment was obtained (GOLDEN GATE, SPECAC, England)). They were allowed to dry and then immersed in G-nHA suspension for 5 min one-by-one. After that, the scaffolds were put on a waxed paper, allowed to freeze for 24 h at – 20 ℃, and finally freeze-dried for 24 h (MVP 12, WONDANG-DONG, SEO-GU, INCHEON, Korea). Subsequently, the freeze-dried PLA/G-nHA scaffolds were cross-linked by immersing in 1% glutaraldehyde (Merck, Germany) for 6 h. The samples were washed twice and allowed to dry under a laminar flow bench.

### Preparation of PLA/G-nHA/PRP scaffolds

Approximately 5 mL of blood was collected in sodium citrate anticoagulant tubes from the rats. The collected blood from rats was centrifuged at 2400 rpm for 10 min to separate the plasma fraction from the red blood cells. The plasma was transferred to 1.5 ml microtubes and further centrifuged at 3600 rpm for 15 min to concentrate the platelets [28]. The upper 2/3 of the liquid (supernatant platelet-poor plasma (PPP)) was thrown away, and the remnant was PRP. Almost 1.5 mL of PRP from 10 mL of blood was obtained. The number of platelets in fresh PRP counted by a cell counter machine, was about 7.28 ± 0.04 times higher than in plasma samples.

The PLA/G-nHA scaffolds were first washed with PBS, and subsequently, immersed in PRP in a testing tube one-by-one on a shaker at ambient temperature for 2 min. The scaffolds were then put on a waxed paper and placed at – 20 ℃ for 24 h. Next, the frozen samples were freeze-dried (MVP 12, WONDANG-DONG, SEO-GU, INCHEON, Korea). The freeze-dried PLA/G-nHA/PRP scaffolds were stored at 4 ℃ until use.

### nHA powder characterizations

The nHA was analyzed by XRD technique to ensure about the phases exist within the powder. The analysis was conducted at 35 kV and 30 mA (Cu Kα radiation with *λ* = 1.5405980 Å). The scanning angle (2*θ*) was from 5° to 80° at a step size of 0.06° (Bruker, D8-advance). Moreover, the morphology of nHA powder was studied using scanning electron microscopy (SEM; TESCAN MIRA3 LMU), and the size distribution was measured for 400 particulates using ImageJ software.

### Structural characterization of scaffolds

To identify the reliability of the printing technique, SEM images of the printed PLA scaffolds were taken and strut thickness and pore dimensions were measured using ImageJ software. The actual pore size and strut dimension of the PLA printed scaffolds were compared with the expected pore size and strut thickness defined in the CAD model. Furthermore, the structure of PLA/G-nHA, and PLA/G-nHA/PRP scaffolds were also observed using SEM (Philips XL30, Netherland).

### Evaluation of physical and mechanical properties

The density of the PLA scaffolds was calculated by weighing the scaffolds and measuring the dimensions, which was subsequently used to estimate the amount of porosity by Eq. 1:

1 $$P\%=\left(1-\frac{{\rho }_{\mathrm{s}}}{{\rho }_{\mathrm{b}}} \right)\times 100.$$

In the above equation, *ρ*_s_ denotes the scaffold density, *ρ*_b_ is the bulk material (PLA without porosity) density, and *P*% signifies the porosity percentage.

The compressive behavior of the fabricated test samples was conducted at ambient temperature using a universal testing machine (UTM, Santam Co., Iran). The displacement speed was set at 1.0 mm/min. Three specimens were tested from each group having a height to diameter ratio of 1.5, 1.25, and 1 (diameter: 7.6 mm, and heights: 11.4, 9.5, and 7.6 mm). The stress–strain curves were drawn by dividing the force to cross-sectional area, and the displacement to initial length. The ultimate compressive strength was then determined considering the maximum stress on the curve, and the compressive modulus was obtained by calculating the elastic region slope from the stress–strain curve of the scaffolds during compression.

### Evaluation of biodegradability

To evaluate the biodegradability of the fabricated scaffolds, simulated body fluid (SBF) was made based on [62], by consecutively dissolving NaCl, NaHCO_3_, KCl, K_2_HPO_4_.3H_2_O, MgCl_2_.6H_2_O, HCl, CaCl_2_, and Na_2_SO_4_ in distilled water. Then, the buffering of the solution was done to pH = 7.4 at 36.5 °C by Tris and 1 M HCl. The compounds used to prepare SBF were all purchased from Merck, Germany. The SBF solution has ion concentrations similar to those of human blood plasma (Na^+^:142.0, K^+^:5.0, Mg^2+^:1.5, Ca^2+^:2.5, Cl^−^:147.8, HCO_3_^−^:4.2, HPO_4_^2−^:1.0, and SO_4_^2−^:0.5 (mM)). To do this test, the scaffolds were first weighed (W_i_) and subsequently immersed in SBF solution. The samples were then put in a thermostatic oven (Behdad Company, Iran) at 37 ± 0.5℃ for 12 weeks. The weighing of the samples was conducted (W_f_) once a week along with refreshing the SBF with new solution. The weight change percentages were calculated based on the following equation:

2 $$W\%=\frac{{W}_{\mathrm{i}}-{W}_{\mathrm{f}}}{{W}_{\mathrm{i}}} \times 100.$$

### Cell culture

Pre-osteoblast MC3T3-E1 cell line (Mouse C57BL/6 calvaria, ECACC, Sigma-Aldrich, Sweden) was cultured in complete Dulbecco's Modified Eagle's medium or DMEM [63] (DNAbioTech; Iran). A complete medium of DMEM was prepared by adding 10 vol.% FBS (Gibco Life Technologies; USA), penicillin (100 IU), and streptomycin (100 μg/ml), and 2 mM l-glutamine (Gibco Life Technologies; USA). The cells were kept in an incubator (5% CO_2_ and 95% humidity) at 37 °C. Routine passaging was conducted on flasks by throwing away the old culture medium, PBS rinsing, trypsinization, centrifugation, and transferring to new flasks having fresh complete medium.

### MTT assay

For MTT test, medium extracts were prepared by incubating the scaffolds in complete culture medium for 72 h at 37℃ in a humidified atmosphere having 5% CO_2_. First, the samples were sterilized using ultra violet (UV) light; 10 min each side of the scaffolds. Two scaffolds of each group were individually immersed in 1.5 mL of medium in a 24-well plate. The PLA/G-nHA/PRP scaffolds were first submerged in CaCl_2_ 20% for 15 min to activate the freeze-dried PRP [64] and then they were immersed in culture medium. The MC3T3-E1 cell line was plated at 5 × 10^3^ cells/well in a 96-well plate and cultured to reach the confluency of 80% (during 24 h). After that, the medium extracts were added and the cells were incubated for 3, 5 and 7 days. Cell proliferation at these time points was assessed by MTT [3-(4, 5-dimethylthiazol-2-yl)-2,5-diphenyltetrazolium bromide] cell proliferation kit (Cell Growth Determination Kit, Sigma Life Science) following the manufacturer’s recommendations. Briefly, 10 μL of MTT solution was put into each well, and the plate was incubated at 37 °C for 4 h. Subsequently, 100 μL of isopropanol-hydrochloric acid solution (0.04 N) was added to each well to dissolve the insoluble formazan formed. The optical density (OD) was measured at 570 nm by a microplate ELISA reader (Synergy H1 Hybrid Multi-Mode Microplate Reader, BioTek, USA).

### Cell adhesion

The cell ability to adhere on the scaffold surfaces was studied using SEM (SERON Technology, South Korea). First, 3 × 10^3^ MC3T3-E1 cells were seeded on the PLA, PLA/G-nHA, and activated PLA/G-nHA/PRP scaffolds. After 72 h incubation time, the adhered cells were fixed with 4% paraformaldehyde (4 °C, 15 min), washed and dried. The scaffolds were then sputter-coated and SEM images were taken at different magnifications in the secondary electron (SE) mode at an accelerating voltage of 25 kV.

### Mineralization

The mineralization (the calcium matrix formation) was evaluated after incubation for 10 days by Alizarin Red staining. First, MC3T3-E1 cells were seeded on the PLA, PLA/G-nHA, and activated PLA/G-nHA/PRP scaffolds at a density of 5 × 10^3^ cells/well in 48-well plates. Complete medium along with osteogenic factors including ascorbic acid, 50 µg/ml, and β-glycerophosphate, 10 mM (Sigma-Aldrich, USA) was used for culturing. After incubation time, the cells on the scaffolds were first fixed by 4% paraformaldehyde (4 °C, 15 min) and stained with 1% (w/v) Alizarin Red in dark for 40 min. At the end, the samples were washed with PBS to remove excessive unattached stain. The scaffolds were imaged and compared with the stained as-fabricated scaffolds (before cell culture as controls).

### In vivo experiments

#### Animals

In the present study, 34 female *Wistar* rats, weighing 250 (± 20) g were used. Standard conditions (20 ℃ temperature under 12/12 h periods of light/darkness) were prepared to maintain adult female rats. Animals were fed and watered ad libitum, and were divided into four groups as indicated in Table 4. All experiments were performed according to the National Institutes of Health guidelines for care and use of animals for scientific purposes.

**Table 4:** Details of animal groups

Group number	Group name	Transplanted specimen	Number of implanted rats for each implantation time
1	Defect	No scaffolds	2
2	PLA	Polylactic acid scaffolds	5
3	PLA/G-nHA	Polylactic acid scaffolds infilled by gelatin-nano-hydroxyapatite	5
4	PLA/G-nHA/PRP	Polylactic acid scaffolds infilled by gelatin-nano-hydroxyapatite and coated by platelet-rich plasma	5

#### Scaffold implantation

**Fig. 13:**
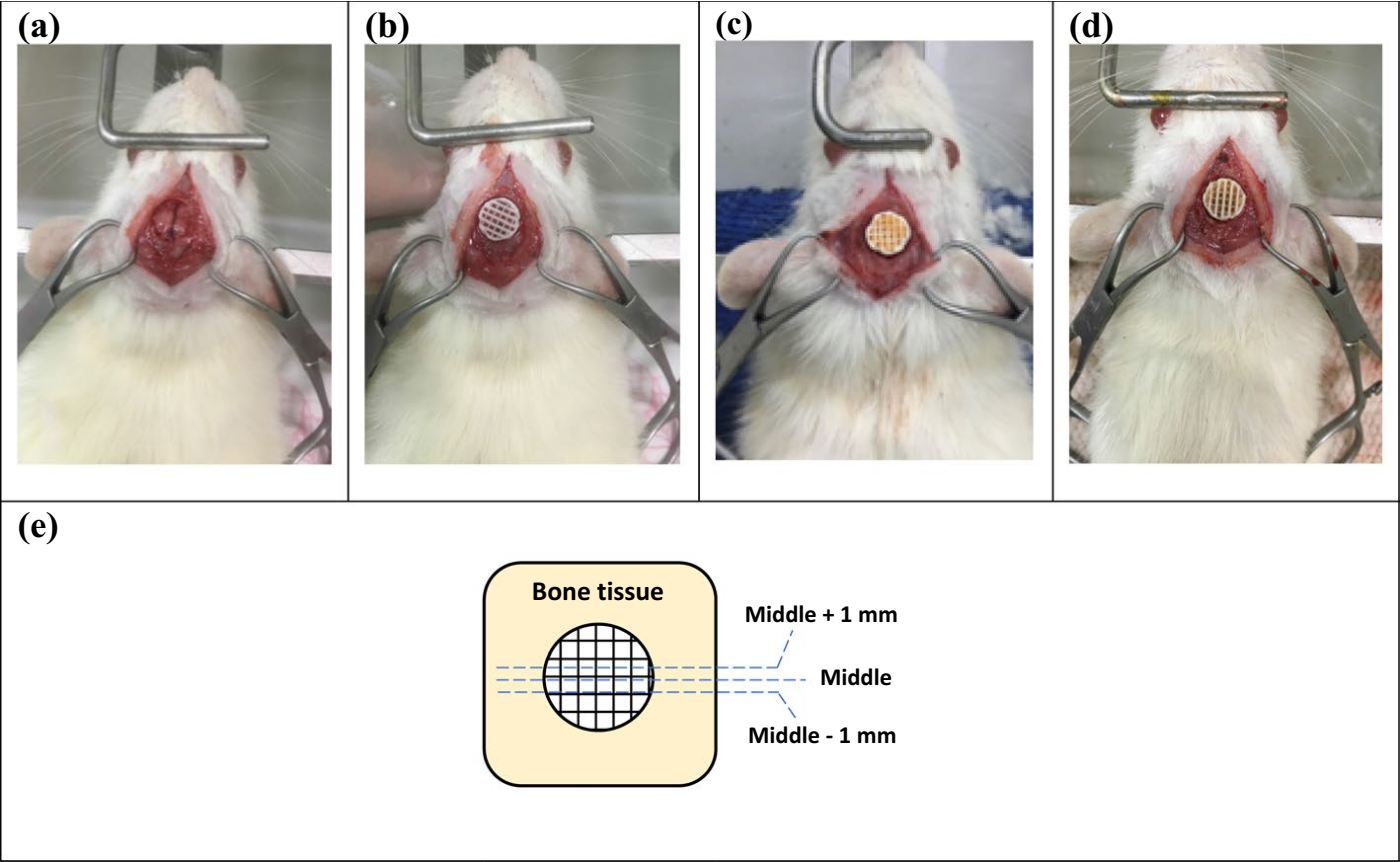
Implantation site and the implanted scaffolds in related groups: **a** defect only, **b** PLA, **c** PLA/G-nHA, **d** PLA/G-nHA/PRP, and **e** studied bone sections in histology

Rats were anesthetized using a mixture with the volume ratio of 8:2 from ketamine hydrochloride and xylazine hydrochloride at 1 mL/kg by intra-peritoneal injection. The implantation of scaffolds was done based on [65]. Briefly, in order to make a midline incision, first the head of the rat was fixed in a stereotaxic instrument, then the hair was cut and disinfected. To expose the calvaria full extent, subperiosteal tissue was dissected bilaterally. By using a surgical trephine bur, one calvarial through-and-through osteotomy was made in the dorsal portion of the parietal bone midsagittal suture under irrigation with sterile normal saline. Eventually, the surgery site was sutured and the bone healing was analyzed after 8 and 12 weeks. The present research was approved by the Ethics Committee of the Faculty of Medicine, Semnan University of Medical Sciences, Iran (Ethic code: IR.SEMUMS.REC.1398.011). Figure 13 also shows the studied group with different scaffolds.

### Histological analysis

After 8 and 12 weeks, the defect sites and their surrounding bone were harvested. The 10% neutral buffered formalin solution was used to fix the collected samples. Then, to complete the decalcification, after washing the samples, they were put in 10% formic acid solution and dehydrated in serially increasing alcohol [66]. The dehydrated samples were embedded in paraffin and 5-µm sections were provided based on Fig. 13e and stained with hematoxylin and eosin (H&E). A light microscope was used to assess the histology slides. Furthermore, after harvesting the defect sites, photos were taken and the macroscopically filled area by new bone was calculated in percent using ImageJ software.

### Serum biochemistry

To assess the systemic influence of scaffolds, a serum biochemistry analysis was performed. At the time of killing at 8 or 12 weeks after the implantation, a blood sample was obtained from the heart of each rat. Approximately 5 mL of blood was collected from each rat, and centrifuged at 3000 rpm for 10 min to acquire the blood serum. Liver enzymes (ALT and AST) activity was analyzed according to the manufacturer's recommendation (Paadco, Golestan Technology Park, Iran).

### Osteocalcin detection

As a bone formation marker, the osteocalcin level was measured in the serum of blood samples collected at the time of killing (8 and 12 weeks after the implantation), using a sandwich ELISA method (rat osteocalcin/bone gamma-carboxyglutamic acid containing protein (OT/BGLAP) ELISA Kit; ZellBio, Germany) following the manufacturer instruction.

### Statistical analysis

Minitab V17 software was used to do the statistical analyses (ANOVA). The confidence level was considered to be 95% (α = 0.05) in all analyses, and Tukey pairwise comparison was conducted to compare the significant differences among the studied groups.

## Data Availability

The data used and/or analyzed during the present study are available from the corresponding author on reasonable request.

## Abbreviations

3D: Three dimensional
ALT: Alanine aminotransferase
ANOVA: Analysis of variance
AST: Aspartate aminotransferase
ATR-FTIR: Attenuated total reflection-Fourier transform infrared
BTE: Bone tissue engineering
CAD: Computer-aided design
E: Elastic modulus
ECM: Extracellular matrix
FDA: Food and Drug Administration
FDM: Fused deposition modeling
G: Gelatin
HA: Hydroxyapatite
H&E: Hematoxylin and eosin
MSC: Mesenchymal stem cells
PCL: Polycaprolactone
PLA: Polylactic acid
PLGA: Polylactic-co-glycolic acid
PPP: Platelet-poor plasma
PRP: Platelet-rich plasma
SBF: Simulated body fluid
SEM: Scanning electron microscopy
STDV: Standard deviation
UCS: Ultimate compressive strength
VEGF: Vascular endothelial growth factor
XRD: X-ray diffraction
